# The mediating roles of coping styles and resilience in the relationship between perceived social support and posttraumatic growth among primary caregivers of schizophrenic patients: a cross-sectional study

**DOI:** 10.1186/s12888-021-03058-9

**Published:** 2021-01-26

**Authors:** Chen Wu, Yaping Liu, Songcui Ma, Guojian Jing, Wei Zhou, Lei Qu, Zitong Wang, Mei Cheng, Yulong Wu

**Affiliations:** 1 School of Nursing, Binzhou Medical University, No.346 Guanhai Road, Yantai, Shandong Province China; 2 School of Nursing, CB 7460, University of North Carolina at Chapel Hill, Chapel Hill, USA; 3 Yantai Yuhuangding Hospital, No.20 Yuhuangding East Road, Yantai, Shandong Province China; 4 Mental Health Center of Shandong Province, No.49 Wenhua East Road, Jinan, Shandong Province China; 5 Department of Pathogenic Biology, Binzhou Medical University, No.346 Guanhai Road, Yantai, Shandong Province China

**Keywords:** Caregivers, Coping skills, Posttraumatic growth, Resilience, Schizophrenia, Social support, Serial-multiple mediation model

## Abstract

**Background:**

Despite the substantial burden of caring schizophrenic patients, primary caregivers can also experience posttraumatic growth (PTG) which may buffer their negative experience. Influencing factors of PTG and their functional pathways among primary caregivers of schizophrenic patients remain unclear. This study is designed to test the simple and serial mediating roles of coping styles and resilience in the relationship between perceived social support and PTG among those primary caregivers.

**Methods:**

A cross-sectional study was conducted from October 2018 to January 2019, and 365 primary caregivers (self-reported) of schizophrenic patients were analyzed. Measures used to assess their perceived social support, coping styles, resilience, and PTG were the Perceived Social Support Scale, the Simplified Coping Style Questionnaire, the Connor-Davidson Resilience Scale, and the Posttraumatic Growth Inventory, respectively. Structural equation modeling was used to run the analysis.

**Results:**

The average scores of PTG (range: 0–5), perceived social support (range: 1–7), positive coping style (range: 0–3), negative coping style (range: 0–3), resilience (range: 0–4) reported by primary caregivers was (2.91 ± 0.99), (4.80 ± 1.26), (1.79 ± 0.65), (1.49 ± 0.56), and (2.46 ± 0.66), respectively. The fitness indices of measurement and structural models were satisfactory. Three indirect pathways totally explained 55.56% variance of the PTG. The indirect effect of positive coping style between perceived social support and PTG was 0.20 [95% confidence interval (CI) 0.05 to 0.37], and this simple mediation pathway explained 27.78% variance of PTG. The indirect effect of resilience between perceived social support and PTG was 0.11 [95% CI 0.01 to 0.20], and this simple mediation pathway explained 15.28% variance of PTG. The indirect effect of positive coping style and then resilience between perceived social support and PTG was 0.09 [95% CI 0.01 to 0.17], and this serial mediation pathway explained 12.50% variance of PTG.

**Conclusions:**

Both simple and serial mediation roles of positive coping style and resilience are established in the relationship between perceived social support and PTG among primary caregivers of schizophrenic patients. Positive coping style and resilience are two important targets for future interventional studies, and interventions on them may bring the synergistic effect on improving PTG.

## Background

Schizophrenia is a disabling group of brain disorders influencing 0.28% of the population worldwide [1], and 0.7% of the population in China [2]. Schizophrenia is not only the traumatic diagnosis for patients, but it also exerts detrimental impacts on their primary caregivers, typically the family members who are involved with the care of the outpatient. However, beneficial changes may simultaneously arise during the struggles of caring for the patients which propel the caregivers to a higher level of psychological functioning than which exists prior to the event, namely the posttraumatic growth (PTG) [3, 4]. For example, evidence indicated that the primary caregivers would perceive positive psychological changes such as improved relationships with others and a deeper appreciation of life [3]. PTG was positively associated with individuals’ quality of life [5], and it was, therefore, important to explore its influencing factors for developing interventional programs to promote the transition from struggles to PTG among primary caregivers of schizophrenic patients.

Perceived social support, defined as the perceived support of individuals through social ties with other individuals, groups and the larger community, was confirmed to play an important and direct role in the development of PTG for primary caregivers of schizophrenic patients [3, 6]. Evidence indicates that more complicated, indirect pathways may exist between perceived social support and PTG.

First, pooled evidence corroborated that perceived social support would optimize the coping ability of schizophrenia patients’ primary caregivers against stress and adversity [7]. Coping styles including positive and negative coping style are defined as the pattern of behavior that is predominantly adopted by individuals when encountering unexpected situations [8] and have been consistently confirmed to be associated with PTG in studies. For example, studies found that positive coping style was positively related to PTG in parents of children with autism [9, 10] and parents of cerebral palsy children [11]. Second, resilience, defined as the ability to adapt positively to negative emotions and “bounce back” from traumatic experiences [12], may serve another pathway between perceived social support and PTG among primary caregivers of schizophrenic patients. Evidence indicated that perceived social support was positively associated with the resilience in primary caregivers of schizophrenic patients [13], while improved resilience was identified to be associated with PTG in primary caregivers of patients with other diseases like cancer [5, 14] and children with autism [15]. One recent publication synthesized evidence and confirmed that positive coping style and resilience both played the mediation roles between perceived social support and PTG among primary caregivers of patients receiving hematopoietic stem cell transplantation [16].

In terms of the relationship between coping styles and resilience, as proposed in the adult resilience model, coping styles would influence resilience which would impact individuals’ quality of life [17]. This proposition was confirmed by an empirical study which stated that coping styles significantly predicted resilience among 128 parents of premature infants hospitalized in a neonatal intensive care unit [18].

**Fig. 1:**
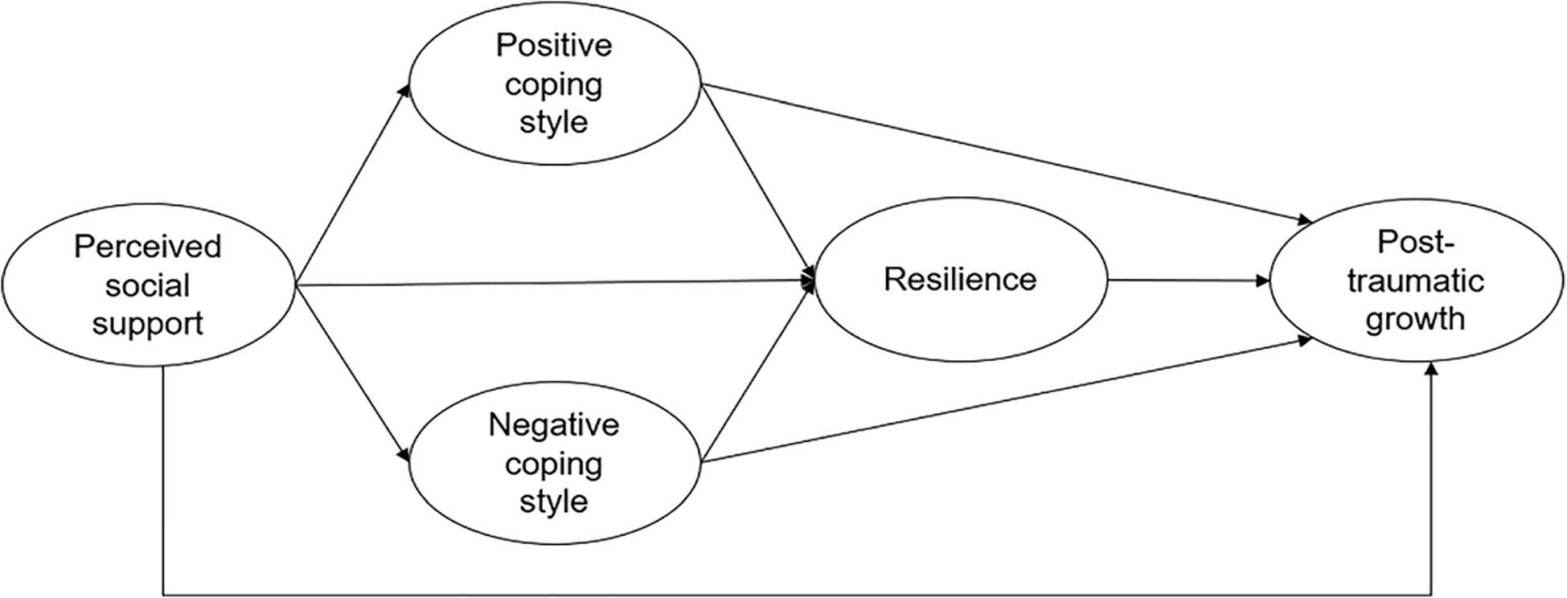
Theoretical framework of the multiple mediation pathways

One theoretical framework demonstrating the relationships between four concepts, i.e., perceived social support, coping styles, resilience, and PTG in primary caregivers of patients was developed from existing studies using the inductive approach, see Fig. 1. However, this multiple-mediation model has not been tested among primary caregivers of schizophrenic patients. To address this gap, in this study, we used data collected from primary caregivers of schizophrenic patients to test three hypotheses.

- *Hypothesis 1*: The relationship between perceived social support and PTG was mediated by coping styles among primary caregivers of schizophrenic patients.
- *Hypothesis 2*: The relationship between perceived social support and PTG was mediated by resilience among primary caregivers of schizophrenic patients.
- *Hypothesis 3*: The relationship between perceived social support and PTG was serially mediated by coping styles and resilience among primary caregivers of schizophrenic patients.

The significant findings from this study may provide important targets for developing interventions to accelerate the transition from struggles of providing care to PTG in primary caregivers, which in turn will guarantee the high quality of service they provide to the schizophrenic patients.

## Methods

### Design and participants

This cross-sectional study was conducted in the Mental Health Center of Shandong Province, Jinan, China, and participants were enrolled using a convenience sampling strategy. The ethical oversight of this study was obtained from the Institutional Review Board of Binzhou Medical University (#2018–47). The Institutional Review Board approved a waiver of signed consent and approved a verbal consent as an alternative given (1) the identifiers included in the medical chart were destroyed (i.e., using coding strategies, like yes or no) when collecting data, and (2) the research involved no more than minimal risk to the subjects.

Inclusion criteria for caregivers were: (1) aged 18 years and older; (2) serving as the primary caregiver (self-reported) for the patient diagnosed of schizophrenia by psychiatrists using the Diagnostic and Statistical Manual of Mental Disorders-IV-text revision [19] or the International Classification of Disease-10 [20]; (3) reporting no experience of other traumatic events, e.g., divorce, serious illness in the past 3 months. Exclusion criteria were: (1) taking care of 2 or more family members either with physical or mental illness simultaneously; (2) having a history of mental illness; (3) refusing to participate.

### Measures

#### Basic characteristics

We used a self-administered questionnaire to collect basic characteristics from primary caregivers of schizophrenic patients. The information collected from primary caregivers included gender, age, educational background, per capita income per month, and their relationship with the patients in care. Patients’ characteristics of gender, age, enroll in the public health insurance or not, and times of hospitalization were retrieved from their medical charts.

#### Response variable: PTG

PTG was assessed with the Chinese version Posttraumatic Growth Inventory (PTGI) [21]. The Chinese version scale has 21 items that are distributed into five domains, i.e., personal strength (4 items), new possibilities (5 items), relating to others (7 items), appreciation of life (3 items), and spiritual change (2 items). The scaling scheme of each item ranges from 0 = not at all to 5 = very strongly degree, with the higher score representing the greater PTG. Good psychometric characteristics of the Chinese version PTGI have been confirmed [21], and in this study, the Cronbach’s alpha coefficients of five domains range from 0.75 to 0.85.

#### Explanatory variable: perceived social support

Perceived social support was assessed with the Chinese version perceived social support scale [22]. The Chinese version scale has 12 items aiming to assess how individuals perceive social support from their families (4 items), friends (4 items), and significant others (4 items). Each item is graded on a 7-point Likert format (1 = very strongly disagree to 7 = very strongly agree), with the higher score representing the stronger perceived social support. Good psychometric characteristics of this scale have been confirmed in Chinese populations [22, 23], and the Cronbach’s alpha coefficients of three domains range from 0.88 to 0.93 in this study.

### Mediating variables

#### Resilience

Resilience was evaluated with the Chinese version Connor-Davidson Resilience Scale (CD-RISC) [24]. The Chinese version scale has 25 items that are distributed into three domains: strength (8 items), optimism (4 items), and tenacity (13 items). Each item is scored from 0 = not at all to 4 = all the time, with the higher score indicating the greater resilience. Studies have confirmed the satisfactory psychometric characteristic of Chinese version CR-RISC [24, 25], and in this study, the Cronbach’s alpha coefficients of three domains range from 0.80 to 0.89.

#### Coping styles

The Simplified Coping Style Questionnaire [26] was used to evaluate caregivers’ coping styles. This scale consists of 20 items which are divided into two subscales: positive coping style (12 items) and negative coping style (8 items). Each item is graded on a 4-point scaling scheme ranging from 0 = never to 3 = always. The higher sum score of each subscale presents the more frequent usage of that coping style. The good reliability and construct validity have been confirmed in the Chinese population [26, 27], and the Cronbach’s alpha coefficient was 0.90 for positive coping style and 0.76 for negative coping style in this study.

### Data collection

Data were collected from October 2018 to January 2019. Trained students in our research team reserved the bright, spacious and quiet room in the center to recruit eligible caregivers, introduce our study, obtain dual informed consent (i.e., their own participation and our review of patients’ medical chart) and distribute the questionnaires. Additional supports for participants with presbyopia and illiterate participants would be provided when needed. The questionnaire took approximately 30 min to complete. Although no formal sample size determination was done, this study did follow the rule of thumb when applying structural equation modeling to run analysis, i.e., the sample size should be at least 200.

### Statistical analysis

Descriptive statistics were used to summarize basic characteristics about the primary caregivers and patients in care, and average scores of domains/subscales for four main concepts (i.e., PTG, perceived social support, resilience and coping styles) reported by primary caregivers were tabulated. Student’s t-test and analysis of variance were applied where appropriate to test the associations between basic characteristics of primary caregivers and their PTG. Distributions of items in scales assessing four main concepts were tested using Shapiro-Wilk statistic in IBM® SPSS® Statistics (version 26, IBM Corp., Armonk, NY), and the estimator was finalized as the maximum likelihood estimation given the normal distribution inferred by − 3.0 to 3.0 for skewness and − 8.0 to 8.0 for kurtosis of this sample. Model testing was performed with Amos 23.0.

We used the domain-representative approach to get item parcels for three multi-domain concepts, i.e., perceived social support, resilience and PTG and random assignment approach to get item parcels for positive coping style and negative coping style [28]. We used path analysis approach to evaluate the construct validity across concepts and then tested the structural model after the validation of the measurement model. The significant basic characteristics of caregivers were controlled as covariates in the structural model.

We used Comparative Fit Index (CFI) ≥ 0.90, normed fit index (NFI) ≥ 0.90, Root Mean Square Error of Approximation (RMSEA) ≤ 0.08, and Standardized Root Mean Square Residual (SRMR) ≤ 0.05, along with the χ^2^ and RMSEA 90% Confidence Interval (CI) to assess the goodness of fit for all models [29]. We used 95% bootstrap CI to evaluate the significance of overall indirect effect and separated indirect effect, and the latter was obtained by running “User-defined estimands” in Amos. Two-sided *p*-value < 0.05 was considered statistically significant.

## Results

### Basic characteristics of primary caregivers and schizophrenic patients in care

Of the 368 primary caregivers recruited, 3 were excluded from analysis because entries for all items within each measure of four main concepts were the same. Among 365 caregivers, a slighter higher proportion of caregivers were males, i.e., 53.70%. Most caregivers were middle-aged adults (> 40 years old) (*n* = 232, 63.56%) and clustered in ‘per capita income per month’ higher than 2000 RMB (*n* = 234, 64.11%); the educational background of all caregivers was almost evenly distributed across three categories, i.e., elementary school or below (32.60%), middle or high school (30.68%), and junior college and above (36.71%); the relationships between the caregivers and patients in care included ‘parent’ caregivers, ‘child’ caregivers and ‘spouse’ caregivers, and their proportions were similar. Male caregivers reported significantly higher scores of PTG compared with their counterparts, see Table 1.

**Table 1:** Basic characteristics of primary caregivers of schizophrenic patients (*N* = 365)

Variables	Frequency (%)	PTG (Mean ± SD)	*p*-value
Gender			0.02
Male	196 (53.70)	63.52 ± 18.91	
Female	169 (46.30)	58.39 ± 22.61	
Age			0.47
age ≤ 40 yrs.	133 (36.44)	60.09 ± 20.23	
age > 40 yrs.	232 (63.56)	61.75 ± 21.19	
Education background			0.16
Elementary school or below	119 (32.60)	58.51 ± 22.87	
Middle or high school	112 (30.68)	63.72 ± 19.90	
Junior college or above	134 (36.71)	61.34 ± 19.52	
Per capita income per month			0.24
Income ≤2000RMB^a^	131 (35.89)	59.35 ± 23.39	
Income >2000RMB	234 (64.11)	62.15 ± 19.23	
The relationship with patients in care			0.33
Parent	133 (36.44)	60.97 ± 20.81	
Child	118 (32.33)	59.25 ± 20.69	
Spouse	114 (31.23)	63.31 ± 20.97	

*Note.* ^a^1RMB = 0.14 USD; PTG indicates posttraumatic growth; SD indicates standard deviation

Patients characteristics retrieved from medical chart showed that 54.52% (199/365) of patients were female; the average age of them was (40.15 ± 14.87) years; 77.26% (282/265) of them enrolled in public health insurance; 52.88% (193/365) of them hospitalized twice and more, and 47.12% (172/365) of them hospitalized once.

### Characteristics of four concepts being modeled

In Table 2, the average score of PTG reported by primary caregivers was (2.90 ± 0.99), personal strength was rated highest, followed by appreciation of life, while spiritual change received the lowest score. The average score of perceived social support was (4.80 ± 1.26); perceived social support from families received the highest mean score; similar scores were reported on perceived social support from significant others and friends. The average score of resilience was (2.46 ± 0.66), with the domain of strength receiving the highest score, followed by tenacity, and optimism is the lowest rated domain. The average scores of negative coping style and positive coping style were (1.49 ± 0.56) and (1.79 ± 0.65), respectively.

**Table 2:** Descriptions statistics of the measured variables (*N* = 365)

Concepts and domains/subscales	Number of items	Score range per item	Mean ± SD
Posttraumatic growth	21	0–5	2.91 ± 0.99
Personal strength	4		3.25 ± 1.12
New possibilities	5		2.58 ± 1.16
Relating to others	7		2.80 ± 1.08
Appreciation of life	3		3.14 ± 1.20
Spiritual change	2		2.23 ± 0.64
Perceived social support	12	1–7	4.80 ± 1.26
From families	4		5.23 ± 1.22
From friends	4		4.56 ± 1.45
From significant others	4		4.60 ± 1.43
Resilience	25	0–4	2.46 ± 0.66
Strength	8		2.56 ± 0.80
Optimism	4		2.25 ± 0.75
Tenacity	13		2.47 ± 0.66
Coping styles	20	0–3	n/a
Negative coping style	8		1.49 ± 0.56
Positive coping style	12		1.79 ± 0.65

*Note.* SD indicates standard deviation; n/a indicates not applicable

### Validation of measurement and structural models

The measurement model encompassed 17 item parcels (i.e., observed variables) that were mapped by 5 latent variables, i.e., perceived social support, resilience, negative coping style, positive coping style and PTG. The goodness-of-fit assessment demonstrated that χ^2^ = 343.93, df = 109, *p* < 0.01, RMSEA = 0.08 with 90% CI of (0.07, 0.09), CFI = 0.95, NFI = 0.93, SRMR = 0.05, which supported the adequate fit of the measurement model. The goodness-of-fit assessment of the multi-domain model showed that χ^2^ = 326.75, df = 122, p < 0.01, RMSEA = 0.07 with 90% CI of (0.06, 0.08), CFI = 0.96, NFI = 0.93, SRMR = 0.05, indicating the adequate fit of the structural model.

### Mediating effects of coping styles and resilience in the relationship between perceived social support and PTG

**Fig. 2:**
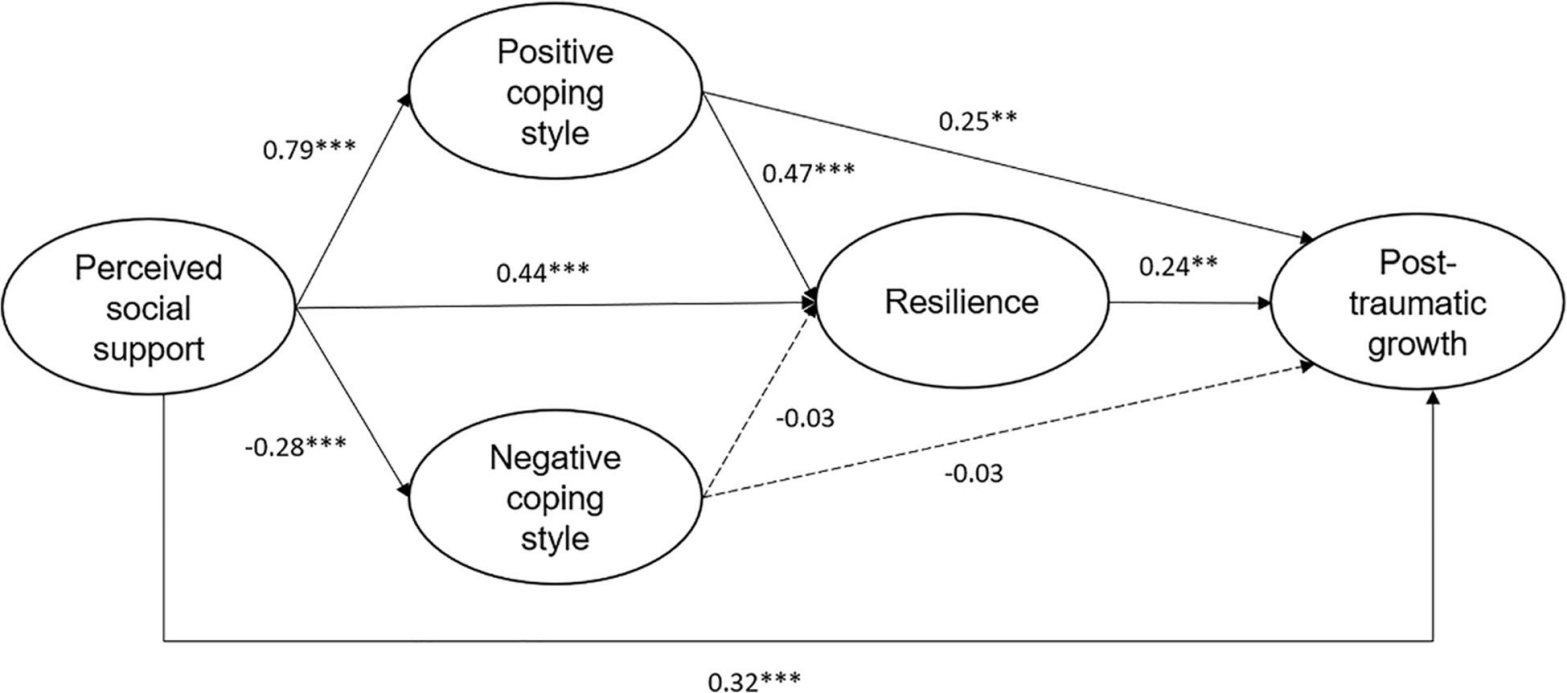
Results of the multiple mediation model. Gender was adjusted for this model testing; all numbers are standardized; ^**^p < 0.01, ^***^p < 0.001

As depicted in Fig. 2, the standardized coefficient of perceived social support on positive coping style was *β* = 0.79, *p* < 0.001 and positive coping style on PTG was *β* = 0.25, *p* < 0.01, and the significant indirect effect of this pathway was 0.20, 95% CI (0.05, 0.37). The standardized coefficient of perceived social support on negative coping style was *β* = − 0.28, *p* < 0.001 and negative coping style on PTG was *β* = − 0.03, *p* > 0.05, the indirect effect of this pathway was not statistically significant.

The standardized coefficient of perceived social support on resilience was *β* = 0.44, *p* < 0.001 and resilience on PTG was *β* = 0.24, *p* < 0.01, the significant indirect effect of this pathway was 0.11, 95% CI (0.01, 0.20).

The standardized coefficient of positive coping style on resilience was *β* = 0.47, *p* < 0.001 and negative coping style on resilience was *β* = − 0.03, *p* > 0.05. The serial mediation pathway of positive coping style and then resilience was significant, with the indirect effect of 0.09, 95% CI (0.01, 0.17), but the serial mediation pathway of negative coping style and then resilience was not significant.

The total indirect effect of three significant pathways was 0.40, 95% CI (0.25, 0.54), and the direct effect of perceived social support on PTG was 0.32, 95% CI (0.14, 0.50). Three indirect pathways totally explained 55.56% variance of the PTG, of which, simple mediation pathways of positive coping style and resilience explained 27.78% variance of PTG and 15.28% variance of PTG, respectively, and the serial mediation pathway explained 12.50% variance of PTG.

## Discussion

Understanding factors influencing PTG in primary caregivers of schizophrenic patients is of great importance for developing innovative interventions to promote their quick transition from possibly negative experiences of providing care to PTG. Previous studies corroborated that perceived social support would directly influence the PTG in primary caregivers of schizophrenic patients [3, 6]. Implied by studies exploring influencing factors of PTG among caregivers of many diseases or conditions, we developed a multiple-mediation model between perceived social support and PTG and tested propositions of it in primary caregivers of schizophrenic patients. The PTG of the overall sample was moderate given its average score of 2.91 grading on 0–5 scoring scheme. Consistent with previous studies [3, 6], the positive association between perceived social support and PTG in primary caregivers of schizophrenic patients was corroborated in this study. Importantly, our research confirmed that positive coping style and resilience would separately mediate the relationship between perceived social support and PTG. There was also a serial mediation pathway between perceived social support and PTG through positive coping style and then resilience.

Hypothesis 1 is partially supported in our study, i.e., only the mediating role of positive coping style for relationship from perceived social support to PTG among primary caregivers of schizophrenic patients is established, and this partial mediation pathway explains 27.78% variance of PTG. This finding provides a new target, i.e., positive coping style to improve PTG of primary caregivers of schizophrenic patients. Positive coping style appears to be low among primary caregivers of schizophrenic patients. The average score of positive coping style is 1.79 grading on the 0–3 scoring scheme, and this score is lower than findings in primary caregivers of patients with mental ill [30] and of patients receiving hematopoietic stem cell transplantation [16]. As positive coping style is positively associated with PTG in primary caregivers of schizophrenia patients, strategies to facilitate them to employ positive coping style are warranted to improve PTG.

This study corroborated the mediating role of resilience for the relationship between perceived social support and PTG among primary caregivers of schizophrenic patients, and this partial mediation pathway explains 15.28% variance of PTG. Resilience is another important target to improve the PTG of interest. The mean score of resilience is 2.46 among primary caregivers of schizophrenic patients, which is close to the median of the 0–4 scoring range (median = 2.00), indicating a moderate degree of resilience. This score was lower than those collected from primary caregivers of older adults [31] and of injured service members [32]. Resilience is positively associated with and PTG in primary caregivers of schizophrenic patients, and resilience-oriented interventions should be employed to enhance their PTG. Such attempts may include cognitive rehearsal and simulation [33], cognitive-behavioral therapy (CBT) [34], and mindfulness training [35], of which the effectiveness has been validated to enhance individuals’ resilience.

The serial mediation pathway between perceived social support and PTG via positive coping style and then resilience among primary caregivers was confirmed in this study, and this pathway explained 12.50% variance of PTG. By drawing scenario in a recent study using data collected from primary caregivers of patients receiving hematopoietic stem cell transplantation [16], this serial mediation pathway offers another important piece of evidence into the literature by revealing other possible pathways in explaining the relationship between perceived social support and PTG among primary caregivers of patients. This finding implies the necessity to integrate factors that have been found to have impacts on the relationship between perceived social support and PTG to offer more comprehensive interventions. Such an attempt may have synergistic effects on improving PTG among primary caregivers of schizophrenic patients.

This study has two limitations. First, although we use an inductive approach to develop the theoretical model depicting the complex, sequential relationships among four concepts (i.e., PTG, perceived social support, resilience, and coping styles), the inherited disadvantage of the cross-sectional design may compromise the assumption of the mediation model, i.e., a causal chain with a specified direction of causal flow. Panel data are warranted to validate findings from this study. Second, we used convenience sampling strategy to enroll participants which could not obtain representative samples. Therefore, the external validity of findings from this study may be limited by the characteristics of schizophrenic patients in care. For instance, more than 50% patients with schizophrenia had two and more times of hospitalization, the findings from this study may not be applicable for primary caregivers of schizophrenic patients with homogenous characteristic in times of hospitalization, e.g., completely newly admitted schizophrenic patients.

## Conclusion

The primary caregivers of schizophrenic patients recruited in this study do not report greater PTG, frequent usage of positive coping style, or greater resilience; our study confirmed the simple and serial mediation roles of positive coping style and resilience in the relationship between perceived social support and PTG. The two mediators, i.e., positive copings style and resilience, provide the targets for developing and launching future intervention to improve PTG in primary caregivers of schizophrenic patients. The finding of serial mediation role of positive coping style and resilience further implies the possible synergistic effect of intervening these two mediators to enhance PTG.

## Data Availability

The data are available from the corresponding authors upon reasonable request.

## Abbreviations

PTG: Posttraumatic growth
PTGI: Posttraumatic Growth Inventory
CD-RISC: Connor-Davidson Resilience Scale
CFI: Comparative Fit Index
NFI: Normed fit index
RMSEA: Root Mean Square Error of Approximation
SRMR: Standardized Root Mean Square Residual
CI: Confidence Interval

## References

[1] Charlson FJ, Ferrari AJ, Santomauro DF (2018). Global epidemiology and burden of schizophrenia: findings from the global burden of disease study 2016. Schizophr Bull.

[2] Huang Y, Wang Y, Wang H (2019). Prevalence of mental disorders in China: a cross-sectional epidemiological study. Lancet Psychiatry.

[3] Balaban OD, Yazar MS, Aydin E (2017). Posttraumatic growth and its correlates in primary caregivers of schizophrenic patients. Indian J Psychiatry.

[4] Hefferon K, Grealy M, Mutrie N (2009). Post-traumatic growth and life threatening physical illness: a systematic review of the qualitative literature. Br J Health Psychol.

[5] Li Y, Qiao Y, Luan X (2019). Family resilience and psychological well-being among Chinese breast cancer survivors and their caregivers. Eur J Cancer Care.

[6] Morton RD, White MJ, Young RM (2015). Posttraumatic growth in family members living with a relative diagnosed with schizophrenia. J Loss Trauma.

[7] Grover S (2015). Pradyumna, Chakrabarti S. coping among the caregivers of patients with schizophrenia. Ind Psychiatry J.

[8] Beutler LE, Harwood TM, Kimpara S (2011). Coping style. J Clin Psychol.

[9] Li LY, Jiang N, Zhao Y (2015). The current status and impact factors of posttraumatic growth in parents of children with autism. Chin J Nurs.

[10] Wang HM, Sun J (2017). The impact of social support and coping styles on posttraumatic growth of in parents of children with autism. Chongqing Med.

[11] Gu YX, Wang X (2016). A prevalence survey on posttraumatic growth and influencing factors of parents of cerebral palsy children. Chin J Health Psychol.

[12] Aburn G, Gott M, Hoare K (2016). What is resilience? An integrative review of the empirical literature. J Adv Nurs.

[13] Lök N, Bademli K. The relationship between the perceived social support and psychological resilience in caregivers of patients with schizophrenia. Community Ment Health J. 2020. 10.1007/s10597-020-00665-w 32591991

[14] Palacio C, Limonero JT (2020). The relationship between the positive aspects of caring and the personal growth of caregivers of patients with advanced oncological illness: Postraumattic growth and caregiver. Support Care Cancer.

[15] Qin XQ, Qu FL, Li YM (2020). Research on the correlation between parents’ posttraumatic growth, mental resilience and family function in children with autism spectrum disorder. Chin J Pract Nurs.

[16] Luo RZ, Zhang S, Liu YH (2020). Short report: relationships among resilience, social support, coping style and posttraumatic growth in hematopoietic stem cell transplantation caregivers. Psychol Health Med.

[17] Haase JE (2004). The adolescent resilience model as a guide to interventions. J Pediatr Oncol Nurs.

[18] Wang CG (2019). The resilience, coping styles and social support of the family members of NICU hospitalized premature infants. Chin General Pract Nurs.

[19] American Psychiatric Association (2000). Diagnostic and statistical manual of mental disorders, DSM-IV.

[20] World Health Organization (2019). The ICD-10 classification of mental and behavioural disorders.

[21] Gao J, Wang M, Deng J (2010). Revision and preliminary application of Chinese version of post-traumatic growth inventory in adolescents experienced the Wenchuan earthquake. Chin Ment Health J.

[22] Huang L, Jiang QJ, Ren WH (1996). The correlation among coping mode, social support and psychosomatic symptoms in patients with cancer. Chin Ment Health J.

[23] Zhou K, Li H, Wei X (2017). Relationships between perceived social support and retention among patients in methadone maintenance treatment in mainland China. Psychol Health Med.

[24] Yu XN, Lau JT, Mak WW (2011). Factor structure and psychometric properties of the Connor-Davidson resilience scale among Chinese adolescents. Compr Psychiatry.

[25] Zhou K, Li J, Li X (2019). Effects of cyclic adjustment training delivered via a mobile device on psychological resilience, depression, and anxiety in Chinese post-surgical breast cancer patients. Breast Cancer Res Treat.

[26] Xie YN (1998). Reliability and validity of the simplified coping style questionnaire. Chinese J Clin Psychol.

[27] Sun P, Sun Y, Jiang H (2019). Gratitude and problem behaviors in adolescents: the mediating roles of positive and negative coping styles. Front Psychol.

[28] Little TD, Cunningham WA, Shahar G (2002). To parcel or not to parcel: exploring the question, weighing the merits. Struct Equ Model.

[29] Byrne BM (2012). Structural equation modeling with Mplus: basic concepts, applications, and programming.

[30] Saeed A (2019). Kiani, Shoaib, et al. caregivers of mentally ill patients: role of coping style and emotional intelligence. Pakistan Armed Forces Med J.

[31] Ong HL, Vaingankar JA, Abdin E (2018). Resilience and burden in caregivers of older adults: moderating and mediating effects of perceived social support. BMC Psychiatry.

[32] Dreer LE, Cox MK, McBrayer A (2019). Resilience among caregivers of injured service members: finding the strengths in caregiving. Arch Phys Med Rehabil.

[33] Clark CM, Gorton KL (2019). Cognitive rehearsal, HeartMath, and simulation: an intervention to build resilience and address incivility. J Nurs Educ.

[34] Padesky CA, Mooney KA (2012). Strengths-based cognitive-behavioural therapy: a four-step model to build resilience. Clin Psychol Psychother.

[35] Galante J, Dufour G, Vainre M (2018). A mindfulness-based intervention to increase resilience to stress in university students (the mindful student study): a pragmatic randomised controlled trial. Lancet Public Health.

